# Field testing of the ICHD-3β and expert opinion criteria for chronic migraine

**DOI:** 10.1186/s10194-016-0678-x

**Published:** 2016-09-19

**Authors:** Huahua Jiang, Yong Deng, Yixin Zhang, Jieli Jin, Xueying Kong, Qiuwen Zhu, Kuiyun Wang, Jiying Zhou

**Affiliations:** 1 Department of Neurology, The First Affiliated Hospital of Chongqing Medical University, 1st You Yi Road, Yu Zhong District, Chongqing, 400016 China; 2 Department of Neurology, The first people’s Hospital of Jintang County, Sichuan, China

**Keywords:** Chronic disease, Humans, International classification of diseases, Migraine disorders/classifications, Migraine disorders/diagnosis

## Abstract

**Background:**

Chronic headache (CrH) occurs commonly in the population, and chronic migraine (CM) accounts for much of the CrH. Diagnostic criteria for CM remain controversial, and this could lead to undertreatment of CM. The purpose of this study was to analyze the clinical profiles of CM and to field test the International Classification of Headache Disorders-3β criteria (ICHD-3β) and Expert Opinion criteria (EO) for CM application.

**Methods:**

We retrospectively reviewed the medical records of CrH patients in our headache clinic during the period. Eligible patients were selected from CrH population based on Silberstein and Lipton criteria (S-L) for CM, and meanwhile fulfilled with migraine days at least 8 days/month. Then we evaluated the characteristics of clinic profiles and outcomes between patients diagnosed CM using ICHD-3β and EO criteria. Field tested the CM criteria Of ICHD-3β and EO.

**Results:**

In a total of 710 CrH patients , 261 (36.8 %) were recruited with CM based on both S-L criteria and fulfilled at least 8 migraine days/month. Be understandable, all the 261 patients met the EO criteria, and only 185 (70.9 %) met ICHD-3β for CM. For the 76 patients who met EO but not ICHD-3β, 70 had atypical migraine attacks (probable migraine, PM), and another 6 had typical migraine attacks but less than a total history of 5 attacks. Although 173 (66.3 %) were concurrent with medication overuse, just one patient overused triptans and none used ergot agents. Clinical features were not significantly different between the ICHD-3β and EO criteria groups (*P* > 0.05), and neither were outcomes of prophylaxis (*P* = 0.966). Total migraine prophylaxis effectiveness was 73 %.

**Conclusion:**

Migraine-specific analgesics are rarely used in China, permitting patients with PM to avail themselves of “migraine days” is a reasonable accommodation for this difficult condition. In our hands, use of the new EO criteria for diagnosis of CM increases the sensitivity and maintains the specificity of decision making, and therefore should be adopted in CM management practice.

## Background

Chronic headache (CrH) refers to a group of headache disorders occurring at least 15 days/month exceeding 3 months, including those associated with medication overuse [1, 2]. Approximately 3 to 5 % of the adult population worldwide have CrH [2]. In practice, the most common form of CrH in patients presenting to headache clinics is CM [2–6]. However, the diagnostic criteria for CM remain controversial.

Transformed migraine (now called chronic migraine, CM) was first proposed by Silberstein and Lipton (S-L) in 1994 [6]. It was classified as CM with or without medication overuse (CM+ and CM-, respectively) [6]. CM criteria were standardized in the Second Edition of the International Classification of Headache Disorders (ICHD-2) in 2004, which required migraine symptoms to be present at least 15 days/month and excluded medication overuse headache (MOH) [7]. Revised criteria (ICHD-2R) published in 2006 modified this, requiring headache at least 15 days/month but also migraine symptoms on at least 8 days. It was still difficult to apply these ICHD-2R criteria in patients with MOH [8].

The International Classification of Headache Disorders, 3rd edition, beta version (ICHD-3β) was published in 2013 [9]. The ICHD-3β criteria for CM require headache at least 15 days/month for least 3 months; it includes migraine with or without aura and believed migraine day relieved by a triptan or ergot derivative, on at least 8 days/month. It also allows CM with and without MOH [9]. However, CrH with probable migraine (PM) are not considered while diagnoses CM by ICHD-3β criteria, not mention the situation that migraine-specific analgesics are still not available worldwide. Thankfully, a paper describing Expert Opinion on diagnostic criteria for CM was published in 2014 [10]. The criteria address PM for treatment of headache with triptans and ergot derivatives [10]. Thus, the purpose of this study was to perform field testing of the ICHD-3β and Expert Opinion criteria (EO) for CM in our patient population.

## Methods

### Study population

A retrospective study was conducted in the headache clinic of the First Affiliated Hospital of Chongqing Medical University in China. Consecutive patient visits presenting with a principal complaint of headache from January 2013 to December 2015 were candidate for this study.

All headache patients with CrH were reviewed by two doctors independently (H.J., Y.D). The diagnosis of CrH was confirmed with at least 15 days/month over a period lasting more than 3 months, which had been defined in our previous study [11]. Eligible patients were selected from CrH if their signs and symptoms fulfilled the criteria for CM according to S-L criteria [6], and with migraine days at least 8 days/month [12]. Patients with other primary CrH (chronic tension-type headache (CTTH), new daily persistent headache (NDPH) and chronic cluster headache et al.) and secondary headaches were excluded [13]. MRI and/or CT scans were performed if clinically necessary. If the headache could not be accurately categorized as either primary or secondary, it was classified as headache not otherwise specified (headache NOS) [11].

The patients were treated empirically with flunarizine (5 mg/day), amitriptyline (25–50 mg/day), topiramate (75 mg/day), metoprolol (25–50 mg/day) or venlafaxine (75 mg/day), based on the specialist’s judgements (JY Zhou) and patient’s preferences. Additionally, overused analgesics were withdrawn in order to address MOH. Patients were informed to discontinue the overused analgesics and meanwhile started prophylactic treatment. Medication responses were classified as significant effectiveness (50 % reduction in headache days of moderate to severe headache), partial effectiveness, and no improvement or worsening symptoms.

### Date collection

All individuals presenting with a principal complaint of headache were interviewed face-to-face by a headache specialist (JY Zhou). The medical history and a detailed structured self-administered questionnaire were completed by 7 doctors (H.J., Y.D,, Y. Z., J.J., X.K., Q.Z., K.W). The questionnaire was validated for headache diagnosis by previous study [5, 11, 14], and included the following components: demographic characteristics, clinical features of headache (onset age, course, location, duration, frequency, intensity, aggravation after physical activity and associated symptoms); history of head injury; past medical history and drug intake for headache; family history. Treatment follow up was then at a 3 to 6-month visit for face-to-face consult, or by telephone interviews, based on previous trials that the migraine prevention should reasonably last for 3–6 months for response and tolerability evaluation [15, 16].

### Standard study protocol and patient consents

Our study protocol was approved by the hospital ethics board. All patients gave their informed consent before collecting headache information.

### Statistical analysis

Statistical analysis was performed using SPSS software, version 20.0 (Chicago, IL, USA). The characteristics of the study population were analyzed using descriptive statistics. Quantitative variables were expressed as mean ± standard deviation (SD). Qualitative variables were expressed as frequency (proportion). The Student’s t test and chi-squared test were used for comparison of clinical parameters between groups. P < 0.05 was considered to be statistically significant.

## Results

**Fig. 1:**
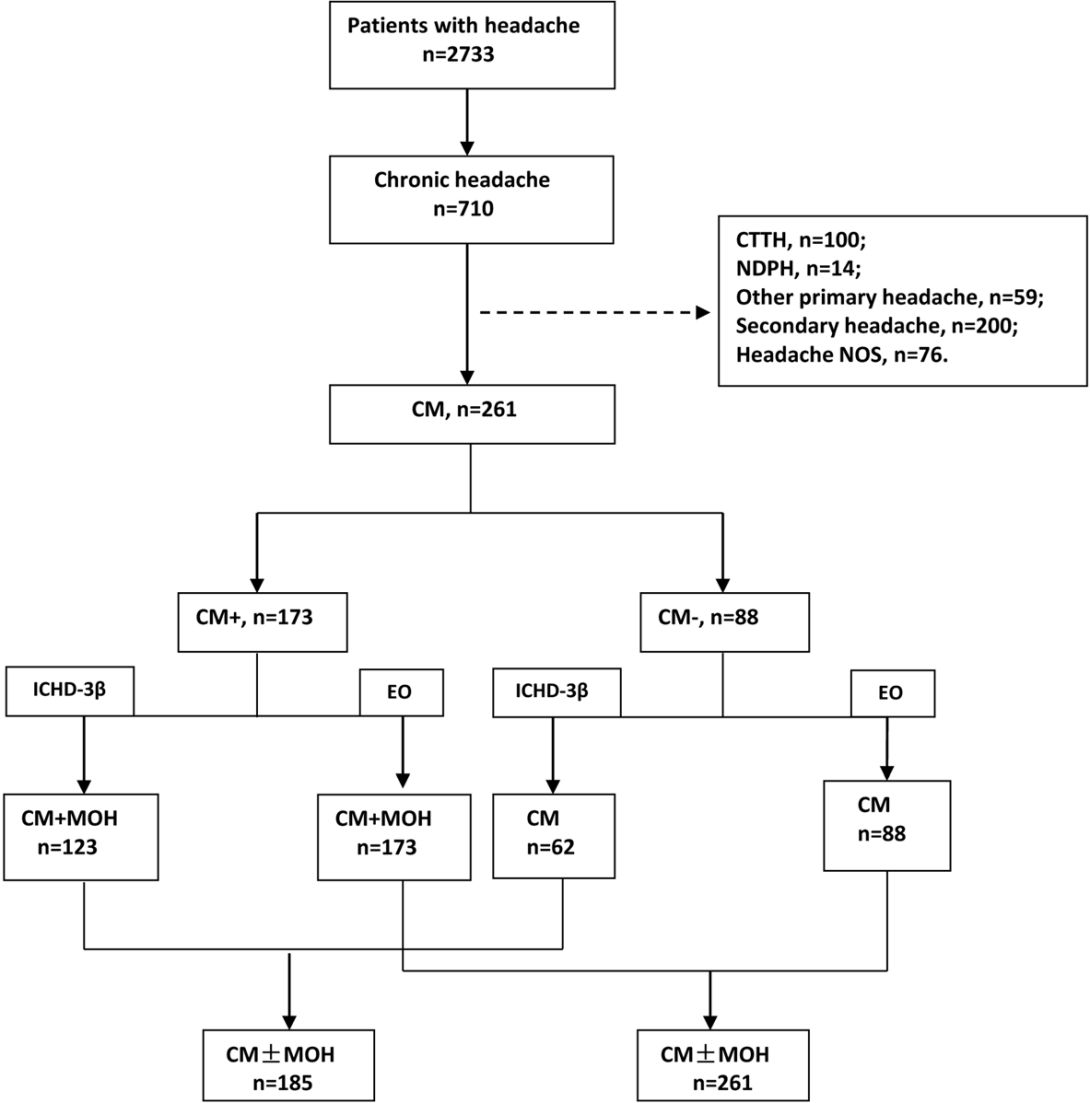
Flow chart of the study. CTTH: chronic tension-type headache; NDPH: new daily persistent headache; Headache NOS: headache not otherwise specified; CM: chronic migraine ; CM- : chronic migraine without medication overuse; CM+: chronic migraine with medication overuse; ICHD-3β: International Classification of Headache Disorders,3rd edition beta version; EO: Expert Opinion criteria; MOH: medication overuse headache

During the study period (January 2013 to December 2015), 2733 patient visits with main complaint of headache were recorded (Fig. 1). A total of 710 (26.0 %) had CrH. Of the CrH patients, 261 (36.8 %) patients were diagnosed as CM based on the S-L criteria, 14.1 % (100/710) had CTTH, 2 % (14/710) had NDPH, 8.3 % (59/710) other primary headache, 28.2 % (200/710) secondary headache and 10.7 % (76/710) headache NOS. CM patients were female predominant (73.2 %) and the mean age was 49.5 years (range from 11 to 83 years).

### The classification of CM using ICHD-3β and EO criteria

Among 261 CM patients, 66.3 % (173/261) overused medication. Of the 88 CM patients without medication overuse, 100 % met EO criteria for CM, but only 62 (70 %) fulfilled ICHD-3β criteria for this diagnosis, as 26 patients had PM being not included in ICHD-3β. Patients with medication overuse were classified as MOH according to ICHD-3β and EO criteria which allow both CM and MOH diagnoses. Of the 173 CM patients with documented medication overuse, 123 (71.1 %) patients fulfilled CM and MOH based on ICHD-3β, other 50 patients were diagnosed with PM. In contrast, all 173 (100 %) patients fulfilled CM and MOH based on EO (Table 1). In total, of 261 CM patients, 70.9 % (185) fulfilled ICHD-3β criteria for CM, and all 261 patients fulfilled EO criteria for CM.

**Table 1:** Classification of patients with CM based on S-L, using ICHD-3β and EO

S-L	ICHD-3β	*n* (%)	EO	*n* (%)
261		185 (70.9 %)		261 (100 %)
CM-, *n* = 88	CM	62 (70.5 %)	CM	88 (100 %)
CM+, *n* = 173	CM + MOH	123 (71.1 %)	CM + MOH	173 (100 %)

*Abbreviations*: *S-L* silberstein and lipton criteria, *CM* chronic migraine without medication overuse, *CM+* chronic migraine with medication overuse, *ICHD-3β* international classification of headache disorders,3rd edition beta version, *EO* expert opinion criteria, *CM* chronic migraine, *CM + MOH* chronic migraine with medication overuse headache

Table 2 summarizes the subtypes of migraine in CM based on ICHD-3β and EO. The subtypes of migraine based on S-L criteria for CM include migraine without aura (MO), migraine with aura (MA), and PM. Among all 261 patients, there were 183 patients meeting the criteria for MO, 8 patients with MA and 70 patients meeting the criteria for PM. Of the 183 MO patients, 177 (96.7 %) patients fulfilled MO based on ICHD-3β criteria for CM, other 6 patients just had less than 5 migraine episodes. 8 (100 %) MA patients fulfilled MA subtypes. The migraine subtypes in EO were in accordance with those in S-L criteria.

**Table 2:** Subtypes of migraine in CM based on ICHD-3β and EO

S-L	ICHD-3β	*n* (%)	EO	*n* (%)
*n* = 261		185 (70.9 %)		261 (100 %)
MO, *n* = 183	MO	177 (96.7 %)	MO	183 (100 %)
MA, *n* = 8	MA	8 (100 %)	MA	8 (100 %)
PM, *n* = 70	–	–	PM	70 (100 %)

*Abbreviations*: *S-L* silberstein and lipton criteria, *MO* migraine without aura, *MA* migraine with aura, *PM* probable migraine, *ICHD-3β* international classification of headache disorders, 3rd edition beta version, *EO* expert opinion criteria

### The clinical features of CM by ICHD-3β and EO criteria

The clinical features of CM patients based on ICHD-3β and EO criteria are shown in Table 3. Demographic profiles showed no significant differences. Mean age was from 48.5 to 49.5 years, and females predominated (73.2–74.1 %). The body mass index averaged from 22.9 to 23.2, within the normal range. The majority of patients (77.8–78.9 %) had not completed high school.

**Table 3:** Clinical features of CM based on ICHD-3β and EO criteria

	ICHD-3β	EO	*P*-value
	*n* = 185	*n* = 261	
Age (years), mean (SD)	48.5 ± 11.4	49.54 ± 11.5	0.364
Gender, *n* (%)			0.837
Female	137 (74.1 %)	191 (73.2 %)	
Male	48 (24.9 %)	70 (26.8 %)	
BMI, mean (SD)	23.2 ± 3.7	22.9 ± 3.6	0.418
Educational level, *n* (%)			0.783
< high school	149 (77.8 %)	206 (78.9 %)	
≥ high school	42 (22.2 %)	55 (21.1 %)	
Frequency of headache days, mean (SD)	23.5 ± 6.4	23.7 ± 6.4	0.750
Frequency of migraine days, mean (SD)	19.9 ± 7.9	20.4 ± 7.9	0.519
Age of onset of migraine, mean (SD)	31.7 ± 12.1	32.1 ± 12.4	0.727
Courses of migraine, mean (SD)	16.8 ± 10.9	17.4 ± 11.7	0.588
Age of onset of CM, mean (SD)	42.3 ± 12.0	42.9 ± 12.1	0.624
Courses of CM, mean (SD)	6.2 ± 8.2	6.8 ± 8.4	0.503
Currently used acute analgesics, *n* (%)			0.811
Yes	154 (83.2 %)	215 (82.5 %)	
No	31 (16.8 %)	46 (17.6 %)	
Category of used analgesics, *n* (%)			0.773
Single analgesics	9 (5.8 %)	14 (6.5 %)	
Combination analgesics	81 (52.6 %)	105 (48.8 %)	
Multiple analgesics	64 (41.6 %)	96 (44.7 %)	
Ergots	0	0	
Triptans	1 (0.6 %)	1 (0.5 %)	0.813
Medication overuse, *n* (%)			0.964
Yes	123 (66.5 %)	173 (66.3 %)	
No	62 (33.5 %)	88 (33.7 %)	
Family headache history, *n* (%)			0.995
Yes	100 (54.1 %)	141 (54.0 %)	
No	85 (45.9 %)	120 (46.0 %)	

*Abbreviations*: *CM* chronic migraine, *BMI* body mass index, *ICHD-3β* international classification of headache disorders,3rd edition beta version, *EO* expert opinion criteria

No headache characteristics were significantly different between the ICHD-3β and EO groups. Frequency of headache was almost identical (23.5–23.7 days per month) by either standard. The courses of migraine were 16.8–17.4 years and the courses of CM were 6.2–6.8 years. The mean age of onset of migraine was 31.7–32.1 years and the mean onset age of CM was 42.3–42.9 years. Most patients (82.5–82.7 %) took analgesic medications. About half (48.8–52.6 %) used combination analgesics, and 41.6–44.7 % of patients were taking multiple analgesics. Just 5.8–6.5 % of patients used simple analgesics. No patients ever used ergots, and only one patient tried triptans. About 54 % of patients had family headache history.

### Treatment outcomes

A total of 250 patients received migraine prophylaxis (flunarizine, *n* = 141; amitriptyline, *n* = 58; topiramate, *n* = 20; metoprolol, *n* = 25; and venlafaxine, *n* = 6). Of these, 62 patients (25 %) were lost to follow-up (flunarizine, *n* = 38; amitriptyline, *n* = 14; topiramate, *n* = 4; and metoprolol, *n* = 5). The causes of lost were of incorrect telephone numbers (*n* = 54), or refusal to follow-up (*n* = 7). One patient discontinued venlafaxine due to nausea.

**Fig. 2:**
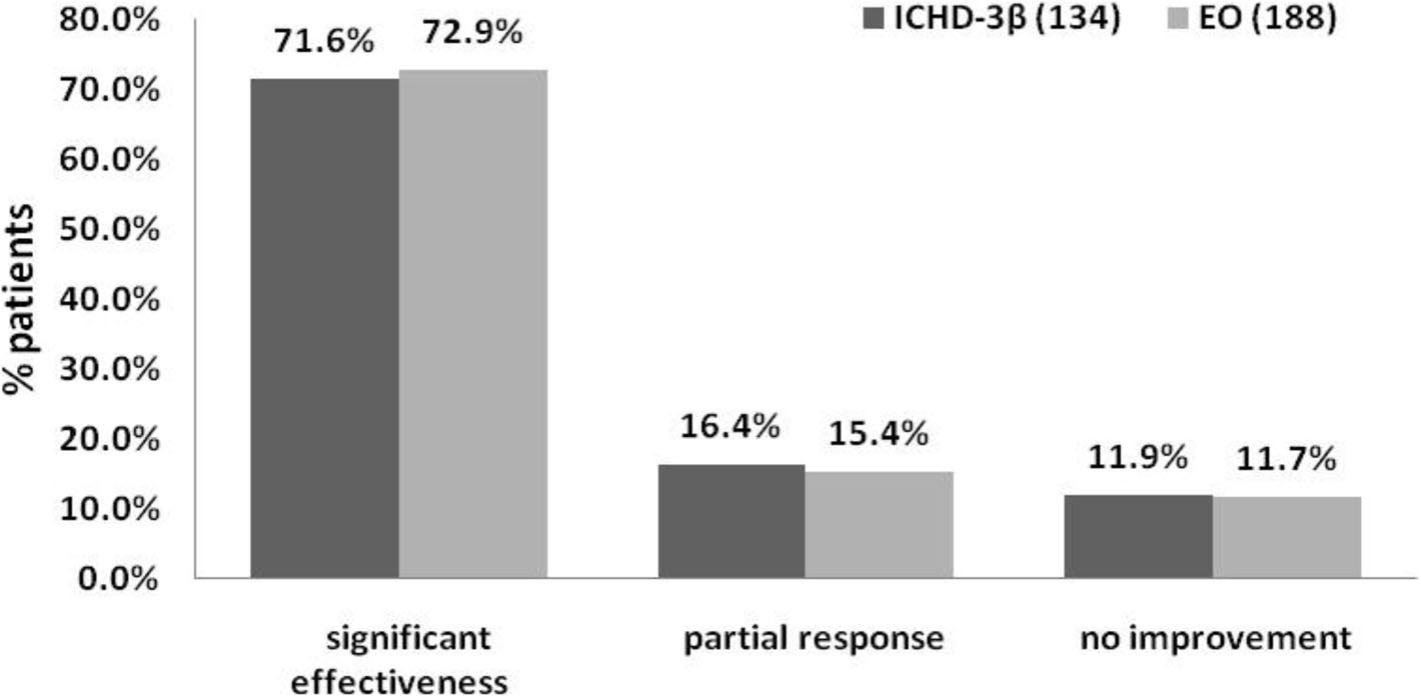
Treatment response of chronic migraine. ICHD-3β: International Classification of Headache Disorders,3rd edition beta version; EO: Expert Opinion criteria

Figure 2 summarizes the treatment response of CM. There was no difference between groups in ICHD-3β and EO (*P* = 0.966). A total of 71.6–72.9 % of the patients had significant reduction of headaches, 15.4–16.4 % of patients had a partial response and the balance (11–12 %) had no improvement which meant these patients had no response to migraine prophylaxis.

## Discussion

This was the first study to evaluate clinical applicability of ICHD-3β and EO criteria for CM. CM is a disabling disorder that is underdiagnosed and undertreated [17]. The prevalence rate of CM is variable. A systematic review in 2003 summarized 12 population-based studies using several definitions for frequent migraine and determined that the global prevalence of CM varied from 0.0 to 5.1 %, with most estimates in the range of 1.4 to 2.2 % [18]. In our study, among 2733 headache patients, a total of 710 patients had CrH. Of the CrH patients, 26.1 % (185/710) patients fulfilled ICHD-3β criteria for CM, but 36.8 % (261/710) patients were diagnosed CM based on EO, and the benefit outcomes who received migraine prophylaxis were identical between the two groups.

In a large US population-based sample, CM accounted for 7.68 % of all migraine cases based on ICHD-2 [19]. Another study, at the Danish Headache Centre, showed that CM accounted for 4.3 % of migraine or tension-type headache based on ICHD-2R [13]. It appears that the proportion of CM varied because of different population sampling techniques or diagnostic criteria.

At present, there is no “gold standard” for diagnosis of CM in clinical practice. ICHD-3β is still considered to be a testing version, and the revised criteria (EO) were proposed in 2014. Table 4 showed the diagnostic criteria of CM.

**Table 4:** Diagnostic criteria for CM according to S-L, ICHD-3β and EO criteria

S-L	ICHD-3β	EO
A. Daily or almost daily (>15 days a month) head pain for >1 month	A. Headache on ≥15 days per month for at least 3 months	A. Headache on ≥15 days per month for at least 3 months
B. Average headache duration of >4 h a day (if untreated)	B. Occurring in a patient who has had at least 5 attacks fulfilling criteria for 1.1 Migraine without aura and/or 1.2 migraine with aura	B. On ≥8 days per month on average ≥4 hours/day for at least 3 months, 1 or more of the following criteria were fulfilled 1. Criteria C and D for 1.1 migraine without aura 2. Criteria B and C for 1.2 migraine with aura 3. Criteria A and B for 1.5 probable migraine
C. At least one of the following: 1. History of episodic migraine meeting any HIS criteria 1.1-1.6 2. History of increasing headache frequency with deceasing severity of migrainous features over at least 3 months 3. Headache at some time meets HIS criteria for migraine 1.1-1.6 other than duration	C. On ≥8 days per month for at least 3 months, one or more of the following criteria were fulfilled 1. Criteria C and D for 1.1 migraine without aura 2. Criteria B and C for 1.2 migraine with aura 3. Believed by the patient to be migraine at onset and relieved by a triptan or ergot derivative	C. Not better accounted for by another ICHD-3 diagnosis
D. Dose not meet criteria for new daily persistent headache (4.7) or hemicrania compounds continua (4.8)	D. Not better accounted for by another ICHD-3 diagnosis.	D. Does not meet criteria for new daily persistent headache (4.7) or hemicrania continua (4.8)

*Abbreviations*: *S-L* silberstein and lipton criteria, *ICHD-3β* international classification of headache disorders, 3rd edition beta version, *EO* expert opinion criteria, *CM* chronic migraine

The three systems of criteria recognize CM with medication overuse. The S-L criteria have been used widely in clinical practice. The criteria required a history of migraine or increasing headache frequency with decreasing severity of migrainous features over at least 3 months, and required a history of transformation. In current opinion, CM is not considered to be a complication of migraine, so the transformation process form episodic attack to continuous pain is not essential condition. Previous study showed that CM can be the initial subtype [20].

The criteria of ICHD-3β require presence of headache on at least 15 days/month for at least 3 months, and establish a diagnostic treatment link to migraine day. It requires migraine with/without an aura or relieved by a triptan or ergot derivative on ≥ 8 days/month. However, it might be too restrictive for application. Firstly, patients with CM often have difficulty recalling their prior migraine attacks as required by item B. Secondly, when analgesic agents are used, migraine features are often not typical or characteristic. Moreover, even migraine-specific analgesics can be effective against other primary headaches, such as cluster headaches, as well as other secondary headaches.

ICHD-3β requires migraine-specific medication as one of the criteria, as patients in some countries took triptans prescribed by their headache clinics, and this could increase the sensitivity of the criteria [21]. A survey in Italy showed that triptans were used by 46.4 % of patients with a previous diagnosis of migraine [17]. Another survey in Latin American showed that 70 % of MOH overused ergotamine, and most of them were migraine sufferers prior to MOH [22]. However, in our population in China, very few patients used triptans or ergot derivatives for treatment of migraine episodes [5, 11, 23]. This may be because that other types of analgesics were not only effective and cheaper than tripans. In this study, just one patient tried triptans, and triptans worked in this patient. The patient had consulted to stop triptans out of overuse. Although PM is considered a migraine subtype by the ICHD-3β, PM days are not counted as migraine days in the CM criteria. Thus some CrH with PM cannot be classified and had no specific diagnosis.

Headache experts proposed revised criteria (EO) in 2014 [10]. The new criteria (1) removed the criterion B that requires CM to occur in a patient with ≥ 5 prior migraine episodes; (2) added PM to C3 regarding treatment and relief of headache by a triptan or ergot agent, and (3) added a requirement that the headache does not meet criteria for NDPH or hemicrania continua. In our study, 70 (26.8 %) patients had atypical migraine episodes, fulfilled the PM [9]. Analyze the 70 PM patients according to the PM criteria, the most criteria not satisfied with was criterion D (associated symptoms), which were up to 70 % (49/70); and criterion B (typical headache duration) was not satisfied in 11.4 % (8/70); criterion C (typical headache features) in 18.6 % (13/70). Migraine attacks might resolve quickly if treated early in the course and before the full symptom complex develops. Additionally, a study in Turkish had reported that migraine characteristics, especially associated symptoms, were less prominent in the CM population, and the phenotype of CM may be associated with the course of classification [24]. So, allowing probable migraines to be a justification for the patient taking a “migraine day”, also serves to include less developed attacks without relying on a migraine-specific treatment response [25].

Among 261 CM patients fulfilling EO criteria, female patients (191, 73.2 %) predominated. This was similar to previous results [5, 26]. The average of age of our patients was 49.5 years, consistent with earlier studies [5, 27]. Most of our patients reported a relatively low level of formal education, which was consistent with our previous study [5]. These patients with CM had an average duration of migraine attacks of 17.4 years, although this is shorter than in another recent study [28]. We note that 82.5 % of our patients took acute analgesics, and 67.6 % had MOH, which was similar to other studies [16, 29, 30]. Finally, the proportion of patients with a positive family headache history was similar to our previous study [5].

Our study was not designed to investigate therapeutic strategies or agents for the treatment of CM, but it did suggest that most patients with CM benefited from 3 to 6-months of treatment with medications for migraine prophylaxis.

## Conclusion

This study was designed to field-test the new EO criteria for CM, beside the ICHD-3β criteria. Migraine-specific analgesics are rarely used in China, accounting for 0.4 % of the agents overused. Given that the ICHD-3β criteria utilize treatment with migraine-specific medication as one of the key data points, it makes this clinical tool less applicable in our clinical setting, and argues for use of the EO criteria. Our results indicate that CrH with PM should be classified into CM based on EO, which will allow more CM sufferers to receive correct diagnosis and adequate treatment. The EO criteria appear to increase the diagnostic sensitivity without lowering specificity, and should be adopted in CM management practice in our setting. Also in our setting, permitting patients with PM to avail themselves of “migraine days” is a reasonable accommodation for treatment of this difficult condition.

### Limitation

This was a retrospective study and selection bias was inevitable. In addition, we treated these patients using medications for migraine prophylaxis. Therefore, further study is needed to establish the efficacy and safety of migraine-specific medications, including different agents, dosage forms, and routes of administration. Moreover, we did not evaluate the disability of these patients, and the factor should be taken into consideration when evaluating the impact of headache. And given that our data were based on patients referred to a specialty clinic for neurological disease, it necessarily will not represent profiles of migraine sufferers in the general population.

### Clinical implication

This study was of major importance for neurologists because chronic migraine (CM) is a disabling disorder that is underdiagnosed and undertreated, and diagnostic criteria for CM remain controversial. In addition, this study analyzed the clinical profiles of CM and to field test the ICHD-3β and Expert Opinion criteria for CM. Migraine-specific analgesics are rarely used in China, permitting patients with probable migraine to avail themselves of “migraine days” is a reasonable accommodation for this difficult condition. The Expert Opinion criteria appear to increase the diagnostic sensitivity without lowering specificity, and should be adopted in CM management practice in our setting.

## Abbreviations

CM: Chronic migraine
CrH: Chronic headache
EO: Expert opinion criteria
ICHD-2: Second Edition of the International Classification of Headache Disorders
ICHD-2R: Revised ICHD-2
ICHD-3β: International Classification of Headache Disorders, 3rd edition, beta version
MA: Migraine with aura
MOH: Medication overuse headache
PM: Probable migraine
